# (5*S*,6*R*)-6-Bromo-6-methyl-5-phenyl-3,4,5,6-tetra­hydro-2*H*-cyclo­penta­[*b*]pyran-7-one

**DOI:** 10.1107/S1600536811038232

**Published:** 2011-09-30

**Authors:** Winai Ieawsuwan, Michael Bolte

**Affiliations:** a Institute of Organic Chemistry, RWTH Aachen University, Landoltweg 1, 52074 Aachen, Germany; b Institut für Anorganische Chemie, J. W. Goethe-Universität Frankfurt, Max-von-Laue-Str. 7, 60438 Frankfurt/Main, Germany

## Abstract

The title compound, C_15_H_15_BrO_2_, was synthesized by a Brønsted acid-catalysed domino electrocyclization-halogenation reaction. The five-membered ring is essentially planar (r.m.s. deviation 0.006 Å) and forms a dihedral angle of 72.7 (3)° with the attached phenyl ring. The six-membered heterocycle adopts a half-chair conformation. The crystal packing is stabilized by a C—H⋯O contact.

## Related literature

For background information, see: Rueping & Ieawsuwan (2009 ▶); Rueping *et al.* (2007 ▶). For the synthesis of the title compound, see: Rueping & Ieawsuwan (2011 ▶). For a comparable compound, see: Liang *et al.* (2003 ▶).
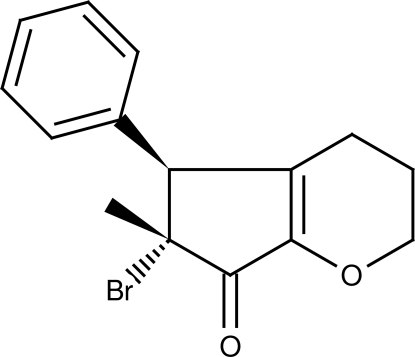


## Experimental

### 

#### Crystal data

C_15_H_15_BrO_2_
*M* *_r_* = 307.18
Orthorhombic, 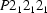

*a* = 9.2217 (11) Å
*b* = 11.5041 (12) Å
*c* = 12.9149 (17) Å
*V* = 1370.1 (3) Å^3^
*Z* = 4
Mo *K*α radiation
μ = 2.99 mm^−1^
*T* = 173 K
0.21 × 0.12 × 0.03 mm

#### Data collection

STOE IPDS II two-circle-diffractometer
Absorption correction: multi-scan (*MULABS*; Spek, 2009 ▶; Blessing, 1995 ▶) *T*_min_ = 0.572, *T*_max_ = 0.916
11129 measured reflections
2407 independent reflections
1849 reflections with *I* > 2σ(*I*)
*R*_int_ = 0.078

#### Refinement

*R*[*F* ^2^ > 2σ(*F* ^2^)] = 0.082
*wR*(*F* ^2^) = 0.217
*S* = 1.03
2407 reflections
163 parameters
H-atom parameters constrained
Δρ_max_ = 1.07 e Å^−3^
Δρ_min_ = −1.13 e Å^−3^
Absolute structure: Flack (1983 ▶), 1009 Friedel pairs
Flack parameter: 0.02 (3)

### 

Data collection: *X-AREA* (Stoe & Cie, 2001 ▶); cell refinement: *X-AREA*; data reduction: *X-AREA*; program(s) used to solve structure: *SHELXS97* (Sheldrick, 2008 ▶); program(s) used to refine structure: *SHELXL97* (Sheldrick, 2008 ▶); molecular graphics: *XP* (Sheldrick, 2008 ▶); software used to prepare material for publication: *SHELXL97*.

## Supplementary Material

Crystal structure: contains datablock(s) I, global. DOI: 10.1107/S1600536811038232/ds2143sup1.cif

Structure factors: contains datablock(s) I. DOI: 10.1107/S1600536811038232/ds2143Isup2.hkl

Supplementary material file. DOI: 10.1107/S1600536811038232/ds2143Isup3.cml

Additional supplementary materials:  crystallographic information; 3D view; checkCIF report

## Figures and Tables

**Table 1:** Hydrogen-bond geometry (Å, °)

*D*—H⋯*A*	*D*—H	H⋯*A*	*D*⋯*A*	*D*—H⋯*A*
C1—H1⋯O31^i^	1.00	2.47	3.282 (9)	138

Symmetry code: (i) 
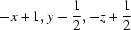
.

**Fig. 1.**
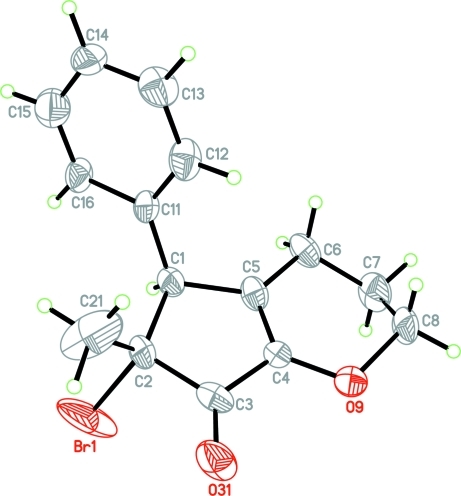
Perspective view of the title compound with the atom numbering scheme and displacement ellipsoids at the 50% probability level.

**Fig. 2.**
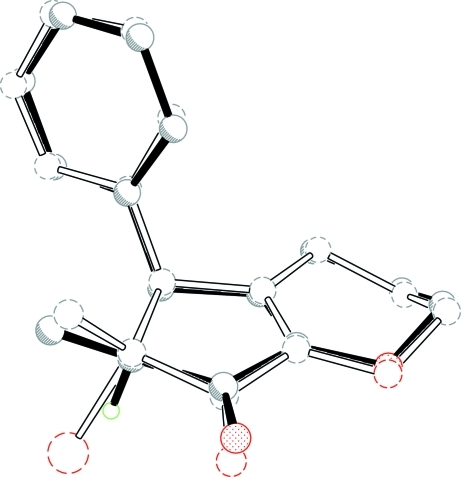
Least-squares fit of the title compound (open bonds) with cis-6-Methyl-5-phenyl-3,4,5,6-tetrahydro-2H-cyclopenta(b)pyran-7-one (full bonds).

## supplementary crystallographic information

### Comment

Trans-4,5-substituted 5-bromocyclopentenone derivatives have been prepared by a organocatalyzed cascade protocol (Rueping & Ieawsuwan, 2009; Rueping *et al.*, 2007). The Brønsted acid catalyzed domino electrocyclization-halogenation reaction provides for the first time, a variety of α-brominated cyclopent-2-enones with a wide substrate scope and with excellent enantioselectivities (Rueping & Ieawsuwan, 2011). Two chiral centers, a tertiary and a quaternary one, can be established during this transformation. The title compound was synthesized for the first time following this reaction and yellow needles suitable for crystal structure determination were obtained.

The five membered ring in the title compound is essentially planar (r.m.s. deviation 0.006 Å) and forms a dihedral angle of 72.7 (3)° with the attached phenyl ring. The six-membered heterocycle adopts a half chair conformation.

A comparable structure, *cis*-6-Methyl-5-phenyl-3,4,5,6-tetrahydro-2*H*-cyclopenta(*b*)pyran-7-one, with an H atom instead of a bromine residue (Liang *et al.*, 2003) has essentially the same conformation (r.m.s. deviation for all C and O atoms 0.183 Å) (Fig. 2).

The crystal packing is stabilized by a C—H···O contact (Table 2).

### Experimental

The title compound has been synthesized as described by Rueping & Ieawsuwan (2011).

### Refinement

All H atoms could be located by difference Fourier synthesis. They were refined with fixed individual displacement parameters [U(H) = 1.2 *U*_eq_(C) or U(H) = 1.5 *U*_eq_(C_methyl_)] using a riding model with C—H ranging from 0.95Å to 1.00 Å.

### Crystal data

**Table:**

C_15_H_15_BrO_2_	*F*(000) = 624
*M**_r_* = 307.18	*D*_x_ = 1.489 Mg m^−^^3^
Orthorhombic, *P*2_1_2_1_2_1_	Mo *K*α radiation, λ = 0.71073 Å
Hall symbol: P 2ac 2ab	Cell parameters from 7315 reflections
*a* = 9.2217 (11) Å	θ = 3.6–25.5°
*b* = 11.5041 (12) Å	µ = 2.99 mm^−^^1^
*c* = 12.9149 (17) Å	*T* = 173 K
*V* = 1370.1 (3) Å^3^	Needle, colourless
*Z* = 4	0.21 × 0.12 × 0.03 mm

### Data collection

**Table:**

STOE IPDS II two-circle-diffractometer	2407 independent reflections
Radiation source: fine-focus sealed tube	1849 reflections with *I* > 2σ(*I*)
graphite	*R*_int_ = 0.078
ω scans	θ_max_ = 25.0°, θ_min_ = 3.5°
Absorption correction: multi-scan (*MULABS*; Spek, 2009; Blessing, 1995)	*h* = −10→10
*T*_min_ = 0.572, *T*_max_ = 0.916	*k* = −13→13
11129 measured reflections	*l* = −15→15

### Refinement

**Table:**

Refinement on *F*^2^	Secondary atom site location: difference Fourier map
Least-squares matrix: full	Hydrogen site location: inferred from neighbouring sites
*R*[*F*^2^ > 2σ(*F*^2^)] = 0.082	H-atom parameters constrained
*wR*(*F*^2^) = 0.217	*w* = 1/[σ^2^(*F*_o_^2^) + (0.1273*P*)^2^ + 1.2132*P*] where *P* = (*F*_o_^2^ + 2*F*_c_^2^)/3
*S* = 1.03	(Δ/σ)_max_ < 0.001
2407 reflections	Δρ_max_ = 1.07 e Å^−^^3^
163 parameters	Δρ_min_ = −1.13 e Å^−^^3^
0 restraints	Absolute structure: Flack (1983), 1009 Friedel pairs
Primary atom site location: structure-invariant direct methods	Flack parameter: 0.02 (3)

### Special details

**Table:**

Experimental. ;
Geometry. All e.s.d.'s (except the e.s.d. in the dihedral angle between two l.s. planes) are estimated using the full covariance matrix. The cell e.s.d.'s are taken into account individually in the estimation of e.s.d.'s in distances, angles and torsion angles; correlations between e.s.d.'s in cell parameters are only used when they are defined by crystal symmetry. An approximate (isotropic) treatment of cell e.s.d.'s is used for estimating e.s.d.'s involving l.s. planes.
Refinement. Refinement of *F*^2^ against ALL reflections. The weighted *R*-factor *wR* and goodness of fit *S* are based on *F*^2^, conventional *R*-factors *R* are based on *F*, with *F* set to zero for negative *F*^2^. The threshold expression of *F*^2^ > σ(*F*^2^) is used only for calculating *R*-factors(gt) *etc*. and is not relevant to the choice of reflections for refinement. *R*-factors based on *F*^2^ are statistically about twice as large as those based on *F*, and *R*- factors based on ALL data will be even larger.

### Fractional atomic coordinates and isotropic or equivalent isotropic displacement parameters (Å^2^)

**Table:**

	*x*	*y*	*z*	*U*_iso_*/*U*_eq_	
Br1	0.4532 (2)	0.30553 (14)	0.06794 (12)	0.1312 (10)	
C1	0.6283 (9)	0.3284 (6)	0.2534 (5)	0.0310 (16)	
H1	0.5988	0.2457	0.2422	0.037*	
C2	0.5646 (11)	0.4039 (7)	0.1618 (5)	0.0404 (19)	
C3	0.4546 (10)	0.4875 (6)	0.2110 (6)	0.0343 (17)	
C4	0.4544 (9)	0.4612 (5)	0.3206 (5)	0.0278 (15)	
C5	0.5450 (10)	0.3752 (6)	0.3462 (5)	0.0307 (17)	
C6	0.5505 (10)	0.3268 (6)	0.4533 (5)	0.0369 (18)	
H6A	0.6408	0.3521	0.4880	0.044*	
H6B	0.5501	0.2408	0.4505	0.044*	
C7	0.4190 (11)	0.3697 (7)	0.5144 (6)	0.046 (2)	
H7A	0.3318	0.3257	0.4929	0.055*	
H7B	0.4348	0.3557	0.5892	0.055*	
C8	0.3945 (10)	0.4988 (7)	0.4959 (6)	0.040 (2)	
H8A	0.3121	0.5260	0.5386	0.048*	
H8B	0.4819	0.5427	0.5170	0.048*	
O9	0.3636 (7)	0.5214 (5)	0.3851 (4)	0.0397 (14)	
C11	0.7886 (9)	0.3324 (6)	0.2644 (5)	0.0289 (16)	
C12	0.8645 (11)	0.4156 (7)	0.3217 (7)	0.040 (2)	
H12	0.8109	0.4724	0.3589	0.048*	
C13	1.0115 (11)	0.4188 (7)	0.3266 (7)	0.045 (2)	
H13	1.0581	0.4778	0.3659	0.054*	
C14	1.0948 (10)	0.3360 (8)	0.2742 (7)	0.045 (2)	
H14	1.1977	0.3366	0.2781	0.054*	
C15	1.0224 (12)	0.2534 (7)	0.2169 (7)	0.047 (2)	
H15	1.0777	0.1984	0.1788	0.057*	
C16	0.8782 (10)	0.2473 (6)	0.2126 (7)	0.0346 (18)	
H16	0.8336	0.1860	0.1748	0.042*	
C21	0.6732 (16)	0.4727 (19)	0.0990 (12)	0.137 (9)	
H21A	0.7434	0.4194	0.0675	0.206*	
H21B	0.7241	0.5274	0.1443	0.206*	
H21C	0.6225	0.5157	0.0444	0.206*	
O31	0.3838 (9)	0.5587 (5)	0.1641 (5)	0.0542 (18)	

### Atomic displacement parameters (Å^2^)

**Table:**

	*U*^11^	*U*^22^	*U*^33^	*U*^12^	*U*^13^	*U*^23^
Br1	0.1929 (19)	0.1099 (10)	0.0906 (10)	0.1023 (12)	−0.1072 (11)	−0.0738 (9)
C1	0.035 (4)	0.035 (4)	0.022 (4)	0.005 (3)	−0.001 (3)	0.001 (3)
C2	0.044 (5)	0.060 (5)	0.018 (3)	0.011 (4)	0.003 (4)	0.004 (3)
C3	0.038 (5)	0.037 (4)	0.028 (4)	−0.006 (3)	−0.009 (4)	0.008 (3)
C4	0.026 (4)	0.033 (3)	0.024 (3)	0.002 (3)	−0.003 (3)	−0.003 (3)
C5	0.039 (5)	0.030 (3)	0.023 (3)	−0.007 (3)	0.004 (3)	0.001 (3)
C6	0.050 (5)	0.040 (4)	0.021 (3)	−0.005 (4)	−0.003 (4)	0.007 (3)
C7	0.052 (6)	0.058 (5)	0.028 (4)	−0.006 (4)	0.011 (4)	0.009 (3)
C8	0.049 (5)	0.053 (4)	0.019 (4)	0.008 (4)	0.010 (4)	0.002 (3)
O9	0.042 (3)	0.046 (3)	0.031 (3)	0.014 (3)	−0.005 (3)	0.001 (2)
C11	0.031 (4)	0.033 (4)	0.023 (3)	−0.001 (3)	0.006 (3)	0.001 (3)
C12	0.047 (6)	0.031 (4)	0.042 (5)	−0.008 (4)	0.009 (4)	−0.010 (4)
C13	0.047 (6)	0.042 (4)	0.046 (5)	−0.019 (4)	−0.001 (4)	−0.005 (4)
C14	0.027 (5)	0.071 (6)	0.038 (4)	−0.003 (4)	0.001 (3)	0.003 (4)
C15	0.051 (7)	0.048 (4)	0.043 (5)	0.004 (4)	0.003 (4)	−0.001 (4)
C16	0.034 (5)	0.034 (4)	0.036 (4)	−0.003 (3)	0.005 (4)	−0.016 (3)
C21	0.077 (10)	0.24 (2)	0.090 (10)	0.074 (12)	0.048 (8)	0.127 (13)
O31	0.088 (5)	0.038 (3)	0.036 (3)	0.023 (3)	−0.015 (3)	0.003 (2)

### Geometric parameters (Å, °)

**Table:**

Br1—C2	1.951 (9)	C8—O9	1.481 (9)
C1—C11	1.486 (11)	C8—H8A	0.9900
C1—C5	1.522 (10)	C8—H8B	0.9900
C1—C2	1.582 (10)	C11—C12	1.398 (11)
C1—H1	1.0000	C11—C16	1.445 (10)
C2—C21	1.512 (17)	C12—C13	1.357 (14)
C2—C3	1.535 (12)	C12—H12	0.9500
C3—O31	1.211 (10)	C13—C14	1.398 (13)
C3—C4	1.447 (11)	C13—H13	0.9500
C4—C5	1.337 (11)	C14—C15	1.377 (13)
C4—O9	1.369 (9)	C14—H14	0.9500
C5—C6	1.491 (9)	C15—C16	1.333 (13)
C6—C7	1.528 (13)	C15—H15	0.9500
C6—H6A	0.9900	C16—H16	0.9500
C6—H6B	0.9900	C21—H21A	0.9800
C7—C8	1.521 (12)	C21—H21B	0.9800
C7—H7A	0.9900	C21—H21C	0.9800
C7—H7B	0.9900		
			
C11—C1—C5	114.6 (6)	H7A—C7—H7B	108.1
C11—C1—C2	115.1 (7)	O9—C8—C7	110.6 (7)
C5—C1—C2	102.0 (6)	O9—C8—H8A	109.5
C11—C1—H1	108.3	C7—C8—H8A	109.5
C5—C1—H1	108.3	O9—C8—H8B	109.5
C2—C1—H1	108.3	C7—C8—H8B	109.5
C21—C2—C3	109.4 (9)	H8A—C8—H8B	108.1
C21—C2—C1	116.3 (8)	C4—O9—C8	112.4 (6)
C3—C2—C1	106.2 (6)	C12—C11—C16	115.0 (8)
C21—C2—Br1	108.6 (10)	C12—C11—C1	124.7 (7)
C3—C2—Br1	105.9 (6)	C16—C11—C1	120.2 (7)
C1—C2—Br1	110.0 (5)	C13—C12—C11	122.9 (8)
O31—C3—C4	129.1 (8)	C13—C12—H12	118.5
O31—C3—C2	124.9 (7)	C11—C12—H12	118.5
C4—C3—C2	106.0 (6)	C12—C13—C14	120.5 (8)
C5—C4—O9	127.3 (7)	C12—C13—H13	119.7
C5—C4—C3	113.3 (6)	C14—C13—H13	119.7
O9—C4—C3	119.4 (6)	C15—C14—C13	117.6 (9)
C4—C5—C6	121.8 (7)	C15—C14—H14	121.2
C4—C5—C1	112.4 (6)	C13—C14—H14	121.2
C6—C5—C1	125.5 (7)	C16—C15—C14	122.9 (9)
C5—C6—C7	109.3 (7)	C16—C15—H15	118.6
C5—C6—H6A	109.8	C14—C15—H15	118.6
C7—C6—H6A	109.8	C15—C16—C11	121.0 (8)
C5—C6—H6B	109.8	C15—C16—H16	119.5
C7—C6—H6B	109.8	C11—C16—H16	119.5
H6A—C6—H6B	108.3	C2—C21—H21A	109.5
C8—C7—C6	110.6 (7)	C2—C21—H21B	109.5
C8—C7—H7A	109.5	H21A—C21—H21B	109.5
C6—C7—H7A	109.5	C2—C21—H21C	109.5
C8—C7—H7B	109.5	H21A—C21—H21C	109.5
C6—C7—H7B	109.5	H21B—C21—H21C	109.5
			
C11—C1—C2—C21	−1.5 (13)	C11—C1—C5—C6	−61.6 (10)
C5—C1—C2—C21	123.2 (12)	C2—C1—C5—C6	173.4 (7)
C11—C1—C2—C3	−123.5 (7)	C4—C5—C6—C7	12.9 (10)
C5—C1—C2—C3	1.2 (8)	C1—C5—C6—C7	−161.6 (8)
C11—C1—C2—Br1	122.4 (6)	C5—C6—C7—C8	−44.3 (9)
C5—C1—C2—Br1	−112.9 (6)	C6—C7—C8—O9	61.9 (10)
C21—C2—C3—O31	53.9 (13)	C5—C4—O9—C8	11.8 (11)
C1—C2—C3—O31	−179.9 (8)	C3—C4—O9—C8	−170.1 (7)
Br1—C2—C3—O31	−63.0 (9)	C7—C8—O9—C4	−44.0 (10)
C21—C2—C3—C4	−126.8 (10)	C5—C1—C11—C12	−30.5 (10)
C1—C2—C3—C4	−0.6 (9)	C2—C1—C11—C12	87.3 (9)
Br1—C2—C3—C4	116.3 (6)	C5—C1—C11—C16	150.4 (7)
O31—C3—C4—C5	178.8 (8)	C2—C1—C11—C16	−91.8 (8)
C2—C3—C4—C5	−0.4 (9)	C16—C11—C12—C13	1.7 (12)
O31—C3—C4—O9	0.4 (13)	C1—C11—C12—C13	−177.4 (8)
C2—C3—C4—O9	−178.9 (6)	C11—C12—C13—C14	−0.9 (14)
O9—C4—C5—C6	4.4 (12)	C12—C13—C14—C15	1.1 (13)
C3—C4—C5—C6	−173.9 (7)	C13—C14—C15—C16	−2.4 (14)
O9—C4—C5—C1	179.6 (7)	C14—C15—C16—C11	3.4 (14)
C3—C4—C5—C1	1.3 (9)	C12—C11—C16—C15	−2.9 (12)
C11—C1—C5—C4	123.5 (7)	C1—C11—C16—C15	176.3 (8)
C2—C1—C5—C4	−1.6 (9)		

### Hydrogen-bond geometry (Å, °)

**Table:**

*D*—H···*A*	*D*—H	H···*A*	*D*···*A*	*D*—H···*A*
C1—H1···O31^i^	1.00	2.47	3.282 (9)	138.

Symmetry codes: (i) −*x*+1, *y*−1/2, −*z*+1/2.
